# Supplementary material for: Folic Acid Supplementation Promotes Hypomethylation in Both the Inflamed Colonic Mucosa and Colitis-Associated Dysplasia
Source: Cancers (Basel). 2023 May 27;15(11):2949. doi: 10.3390/cancers15112949 (PMC10252136; doi:10.3390/cancers15112949)
Supplement: Supplementary file 1 [file cancers-15-02949-s001.zip › cancers-2408420-supplementary.pdf]

Supplemental Table S1. Genes were significantly methylated between 8 mg FA vs. 0 mg FA treated epithelium					
411 genes, 347 Cp	16	344	133		196 genes (264 CpGs)
CpGs 8N vs 0N	RNA 8N Vs 0N	Expressed 0N (sum reads >10)	RNA 8P Vs 8N	RNA 0P vs 0N	CpGs 0P vs 0N
T	Yes(DESeq2)	N	Yes(CuffDiff)	Yes(DESeq2)	0
Slc4a11	Yes(DESeq2)	Y	Yes(CuffDiff & DESeq2)	Yes(CuffDiff & DESeq2)	2
Epha2	Yes(DESeq2)	Y	Yes(CuffDiff & DESeq2)	Yes(CuffDiff & DESeq2)	1
Enc1	Yes(DESeq2)	Y	Yes(CuffDiff & DESeq2)	Yes(CuffDiff & DESeq2)	0
Rab11fip4	Yes(DESeq2)	Y	Yes(CuffDiff & DESeq2)	Yes(CuffDiff & DESeq2)	0
Atp11a	Yes(DESeq2)	Y	Yes(CuffDiff & DESeq2)	Yes(CuffDiff & DESeq2)	0
Zfp703	Yes(DESeq2)	Y	No	Yes(CuffDiff & DESeq2)	0
Rims4	Yes(CuffDiff)	Y	Yes(DESeq2)	Yes(CuffDiff & DESeq2)	1
Lzts1	Yes(CuffDiff)	Y	Yes(CuffDiff & DESeq2)	Yes(DESeq2)	0
Dusp4	Yes(CuffDiff)	Y	Yes(CuffDiff & DESeq2)	Yes(CuffDiff & DESeq2)	3
Spink4	Yes(CuffDiff)	Y	Yes(CuffDiff & DESeq2)	No	1
Tns4	Yes(CuffDiff)	Y	Yes(CuffDiff & DESeq2)	No	2
Fut9	Yes(CuffDiff)	Y	No	Yes(CuffDiff & DESeq2)	2
Neu1	Yes(CuffDiff & DESeq2)	Y	Yes(DESeq2)	Yes(CuffDiff & DESeq2)	2
Thbs1	Yes(CuffDiff & DESeq2)	Y	Yes(CuffDiff & DESeq2)	Yes(CuffDiff & DESeq2)	2
Arntl	No	Y	Yes(DESeq2)	No	2
Accn1	No	Y	Yes(DESeq2)	No	0
Foxg1	No	Y	Yes(DESeq2)	Yes(DESeq2)	0
Il1r2	No	Y	Yes(DESeq2)	Yes(DESeq2)	0
Trim29	No	Y	Yes(DESeq2)	Yes(DESeq2)	1
Gata4	No	Y	Yes(DESeq2)	Yes(DESeq2)	0
Cdh4	No	Y	Yes(DESeq2)	No	1
Zbtb32	No	Y	Yes(DESeq2)	No	1
Tbx4	No	Y	Yes(DESeq2)	Yes(DESeq2)	0
Crybb3	No	Y	Yes(DESeq2)	No	0
Ptch2	No	Y	Yes(DESeq2)	Yes(DESeq2)	2
Mapk10	No	Y	Yes(DESeq2)	Yes(DESeq2)	0
Syt2	No	Y	Yes(DESeq2)	Yes(DESeq2)	0
Hrc	No	Y	Yes(DESeq2)	No	1
Lrp11	No	Y	Yes(DESeq2)	Yes(DESeq2)	0

2310050B05Rik	No	Y	Yes(DESeq2)	Yes(DESeq2)	1
Foxl1	No	Y	Yes(DESeq2)	Yes(DESeq2)	1
Lmx1a	No	Y	Yes(DESeq2)	Yes(DESeq2)	0
Edn2	No	Y	Yes(DESeq2)	Yes(DESeq2)	0
Hkdc1	No	Y	Yes(DESeq2)	Yes(DESeq2)	2
Ccdc39	No	Y	Yes(DESeq2)	Yes(DESeq2)	0
Zfpm1	No	Y	Yes(DESeq2)	Yes(DESeq2)	5
Adrb1	No	Y	Yes(DESeq2)	Yes(DESeq2)	0
Zfp637	No	Y	Yes(DESeq2)	Yes(DESeq2)	1
Gpr146	No	Y	Yes(DESeq2)	No	0
Chchd5	No	Y	Yes(DESeq2)	No	1
Pdzd3	No	Y	Yes(DESeq2)	Yes(DESeq2)	1
Olfm1	No	Y	Yes(DESeq2)	Yes(DESeq2)	1
Gas8	No	Y	Yes(DESeq2)	Yes(DESeq2)	0
Usp2	No	Y	Yes(DESeq2)	Yes(CuffDiff & DESeq2)	0
Fam108a	No	Y	Yes(DESeq2)	No	0
Mon1a	No	Y	Yes(DESeq2)	No	0
Cspg4	No	Y	Yes(DESeq2)	Yes(DESeq2)	0
Palm	No	Y	Yes(DESeq2)	No	0
Phf16	No	Y	Yes(DESeq2)	Yes(DESeq2)	1
Nkx2-3	No	Y	Yes(DESeq2)	No	0
Cntrob	No	Y	Yes(DESeq2)	Yes(DESeq2)	2
Dpep1	No	Y	Yes(DESeq2)	Yes(DESeq2)	1
Cygb	No	Y	Yes(DESeq2)	No	1
Kctd5	No	Y	Yes(DESeq2)	Yes(DESeq2)	0
Sephs1	No	Y	Yes(DESeq2)	Yes(DESeq2)	0
Prkag1	No	Y	Yes(DESeq2)	No	0
Camta1	No	Y	Yes(DESeq2)	Yes(DESeq2)	2
Fgd5	No	Y	Yes(DESeq2)	Yes(DESeq2)	1
Nfatc2	No	Y	Yes(DESeq2)	Yes(DESeq2)	3
Zfp395	No	Y	Yes(DESeq2)	Yes(CuffDiff & DESeq2)	0
Iqsec2	No	Y	Yes(DESeq2)	Yes(CuffDiff & DESeq2)	1
Mst1r	No	Y	Yes(DESeq2)	Yes(DESeq2)	0
Cul4b	No	Y	Yes(DESeq2)	No	2
Chmp1a	No	Y	Yes(DESeq2)	No	1

Tenc1	No	Y	Yes(DESeq2)	Yes(CuffDiff & DESeq2)	0
Liph	No	Y	Yes(DESeq2)	No	2
Atp6v1b2	No	Y	Yes(DESeq2)	Yes(DESeq2)	0
Irs2	No	Y	Yes(DESeq2)	Yes(CuffDiff & DESeq2)	0
Trp53bp2	No	Y	Yes(DESeq2)	Yes(DESeq2)	1
Arhgef16	No	Y	Yes(DESeq2)	No	0
Dip2a	No	Y	Yes(DESeq2)	Yes(CuffDiff & DESeq2)	0
Vamp3	No	Y	Yes(DESeq2)	Yes(DESeq2)	2
Pcyt1a	No	Y	Yes(DESeq2)	Yes(DESeq2)	1
Zadh2	No	Y	Yes(DESeq2)	Yes(CuffDiff & DESeq2)	1
Kank2	No	Y	Yes(DESeq2)	Yes(CuffDiff & DESeq2)	0
Atp8a2	No	Y	Yes(DESeq2)	Yes(DESeq2)	0
Dock6	No	Y	Yes(DESeq2)	Yes(DESeq2)	0
Pecam1	No	Y	Yes(DESeq2)	No	1
Ndrg1	No	Y	Yes(DESeq2)	No	0
Crim1	No	Y	Yes(DESeq2)	Yes(CuffDiff & DESeq2)	0
Tom1l2	No	Y	Yes(DESeq2)	Yes(CuffDiff & DESeq2)	0
Rbms1	No	Y	Yes(DESeq2)	Yes(DESeq2)	2
Arhgap26	No	Y	Yes(DESeq2)	Yes(CuffDiff & DESeq2)	1
L1cam	No	Y	Yes(DESeq2)	Yes(DESeq2)	0
H2afy	No	Y	Yes(DESeq2)	Yes(DESeq2)	1
Csrp1	No	Y	Yes(DESeq2)	No	0
Golph3	No	Y	Yes(DESeq2)	Yes(DESeq2)	1
Tmed2	No	Y	Yes(DESeq2)	No	0
Eps8l3	No	Y	Yes(DESeq2)	Yes(CuffDiff & DESeq2)	0
Gapvd1	No	Y	Yes(DESeq2)	No	0
Sec16a	No	Y	Yes(DESeq2)	No	0
Sec63	No	Y	Yes(DESeq2)	Yes(DESeq2)	0
Nr3c1	No	Y	Yes(DESeq2)	Yes(CuffDiff & DESeq2)	1
Ppp1r15b	No	Y	Yes(DESeq2)	Yes(DESeq2)	1
Cytsa	No	Y	Yes(DESeq2)	No	1
Krt8	No	Y	Yes(DESeq2)	No	1
Btbd17	No	N	Yes(DESeq2)	Yes(DESeq2)	0
Wnt7b	No	N	Yes(DESeq2)	Yes(DESeq2)	0
Rhpn1	No	N	Yes(DESeq2)	No	1

9830001H06Rik	No	Y	Yes(CuffDiff)	Yes(DESeq2)	0
Ccna2	No	Y	Yes(CuffDiff)	Yes(DESeq2)	0
Itpripl2	No	Y	Yes(CuffDiff)	Yes(CuffDiff & DESeq2)	1
Robo2	No	Y	Yes(CuffDiff)	No	0
Fst	No	Y	Yes(CuffDiff & DESeq2)	Yes(CuffDiff & DESeq2)	0
Grhl3	No	Y	Yes(CuffDiff & DESeq2)	Yes(DESeq2)	2
Mycn	No	Y	Yes(CuffDiff & DESeq2)	No	0
Slc26a10	No	Y	Yes(CuffDiff & DESeq2)	Yes(CuffDiff)	0
Phlda3	No	Y	Yes(CuffDiff & DESeq2)	Yes(CuffDiff & DESeq2)	0
Rasl11b	No	Y	Yes(CuffDiff & DESeq2)	Yes(CuffDiff & DESeq2)	0
B3gnt6	No	Y	Yes(CuffDiff & DESeq2)	Yes(CuffDiff)	3
Grasp	No	Y	Yes(CuffDiff & DESeq2)	Yes(DESeq2)	1
Cd79b	No	Y	Yes(CuffDiff & DESeq2)	No	1
Ppfia3	No	Y	Yes(CuffDiff & DESeq2)	Yes(CuffDiff & DESeq2)	0
Zdhhc14	No	Y	Yes(CuffDiff & DESeq2)	Yes(CuffDiff & DESeq2)	1
Chst11	No	Y	Yes(CuffDiff & DESeq2)	Yes(CuffDiff & DESeq2)	1
Sntg2	No	Y	Yes(CuffDiff & DESeq2)	Yes(CuffDiff & DESeq2)	0
Cadm1	No	Y	Yes(CuffDiff & DESeq2)	Yes(CuffDiff & DESeq2)	1
Galm	No	Y	Yes(CuffDiff & DESeq2)	Yes(CuffDiff & DESeq2)	0
Epb4.1l4a	No	Y	Yes(CuffDiff & DESeq2	No	0
Slc41a1	No	Y	Yes(CuffDiff & DESeq2)	Yes(CuffDiff & DESeq2)	0
Pogk	No	Y	Yes(CuffDiff & DESeq2)	Yes(DESeq2)	2
Arhgap24	No	Y	Yes(CuffDiff & DESeq2)	Yes(CuffDiff)	0
Irf4	No	Y	Yes(CuffDiff & DESeq2)	Yes(DESeq2)	0
Lamc2	No	Y	Yes(CuffDiff & DESeq2)	Yes(CuffDiff & DESeq2)	1
Mta1	No	Y	Yes(CuffDiff & DESeq2)	Yes(DESeq2)	1
Znrf3	No	Y	Yes(CuffDiff & DESeq2)	Yes(CuffDiff & DESeq2)	1
Bcl9	No	Y	Yes(CuffDiff & DESeq2)	Yes(DESeq2)	0
Notch1	No	Y	Yes(CuffDiff & DESeq2)	Yes(DESeq2)	0
Pck1	No	Y	Yes(CuffDiff & DESeq2)	Yes(CuffDiff & DESeq2)	1
Cdx1	No	Y	Yes(CuffDiff & DESeq2)	Yes(CuffDiff & DESeq2)	0
Map4k4	No	Y	Yes(CuffDiff & DESeq2)	Yes(CuffDiff & DESeq2)	2
Slc44a4	No	Y	Yes(CuffDiff & DESeq2)	Yes(CuffDiff & DESeq2)	2
Dgkh	No	Y	Yes(CuffDiff & DESeq2)	Yes(CuffDiff & DESeq2)	0
Irx4	No	N	Yes(CuffDiff & DESeq2)	Yes(CuffDiff & DESeq2)	0

Adam18	No	Y	No	No	0
Scn4a	No	Y	No	No	1
Frmpd3	No	Y	No	No	0
Loxhd1	No	Y	No	No	1
Osta	No	Y	No	No	1
Dazl	No	Y	No	No	1
Nupl1	No	Y	No	No	0
Gpr123	No	Y	No	No	0
Tmem17	No	Y	No	No	0
Gm10941	No	Y	No	No	0
Ggn	No	Y	No	No	0
Rxrg	No	Y	No	No	0
Slit1	No	Y	No	No	2
Dlgap3	No	Y	No	No	0
Col4a4	No	Y	No	No	0
Gm5424	No	Y	No	No	0
Proc	No	Y	No	No	0
Slc13a3	No	Y	No	No	0
Tspan10	No	Y	No	No	0
Fam19a5	No	Y	No	No	0
T2	No	Y	No	No	0
Dbc1	No	Y	No	No	0
Lrp1b	No	Y	No	No	0
E130012A19Rik	No	Y	No	Yes(CuffDiff & DESeq2)	0
Ccr7	No	Y	No	No	2
Rasl10b	No	Y	No	No	0
Kcnh1	No	Y	No	No	0
Fmod	No	Y	No	No	1
Slc2a6	No	Y	No	No	0
Krt80	No	Y	No	No	0
Atcay	No	Y	No	No	1
Hipk4	No	Y	No	No	1
2210417A02Rik	No	Y	No	No	1
St8sia5	No	Y	No	No	1
Srcin1	No	Y	No	No	0

Eomes	No	Y	No	No	0
Ldhal6b	No	Y	No	No	1
4833422F24Rik	No	Y	No	No	0
Slc26a6	No	Y	No	No	0
Tusc1	No	Y	No	No	0
Slc36a2	No	Y	No	No	1
Celsr3	No	Y	No	No	0
BC002163	No	Y	No	No	1
Fam65c	No	Y	No	No	0
Eda	No	Y	No	No	0
Vmn2r99	No	Y	No	No	1
4930590J08Rik	No	Y	No	No	1
Abcg8	No	Y	No	No	0
Bend7	No	Y	No	Yes(DESeq2)	0
1200009I06Rik	No	Y	No	No	0
Slc41a3	No	Y	No	No	0
Col11a2	No	Y	No	Yes(DESeq2)	0
Meox1	No	Y	No	No	1
Obscn	No	Y	No	No	3
Ido1	No	Y	No	No	0
Prx	No	Y	No	No	1
Gm885	No	Y	No	No	1
Ikzf4	No	Y	No	No	0
Pde6g	No	Y	No	No	0
Pfn2	No	Y	No	Yes(DESeq2)	1
Fosb	No	Y	No	No	1
Prdm16	No	Y	No	No	1
Cacna1i	No	Y	No	No	0
Slc46a3	No	Y	No	No	2
Pdzd4	No	Y	No	No	0
Evx2	No	Y	No	No	1
Egr3	No	Y	No	No	1
Asf1b	No	Y	No	No	0
Iqsec3	No	Y	No	No	0
Pou3f2	No	Y	No	No	0

Fam83d	No	Y	No	No	1
Fstl3	No	Y	No	No	0
Rps19bp1	No	Y	No	No	0
Jdp2	No	Y	No	No	1
8430419L09Rik	No	Y	No	No	1
Oas3	No	Y	No	No	0
Mtus2	No	Y	No	Yes(DESeq2)	2
Neil2	No	Y	No	No	1
Suox	No	Y	No	No	0
Rilpl1	No	Y	No	No	0
Efemp2	No	Y	No	Yes(DESeq2)	0
Mus81	No	Y	No	No	0
Alg1	No	Y	No	No	1
Kynu	No	Y	No	No	0
2610301B20Rik	No	Y	No	No	0
Dtd1	No	Y	No	No	2
Ror2	No	Y	No	No	1
Hic1	No	Y	No	No	1
Prdx4	No	Y	No	No	0
Thap1	No	Y	No	No	0
Ube2g2	No	Y	No	No	0
Miat	No	Y	No	No	1
Zbtb45	No	Y	No	No	1
Adck1	No	Y	No	No	2
Cxcr7	No	Y	No	Yes(DESeq2)	2
Plxna4	No	Y	No	No	1
Cdk18	No	Y	No	Yes(CuffDiff & DESeq2)	0
Trib2	No	Y	No	No	0
Katnal1	No	Y	No	No	1
Myo1g	No	Y	No	No	0
Nufip1	No	Y	No	Yes(DESeq2)	1
Grid1	No	Y	No	No	0
2410131K14Rik	No	Y	No	No	0
Anpep	No	Y	No	No	1
Vmn2r112	No	Y	No	No	1

Ncdn	No	Y	No	No	0
Rin3	No	Y	No	No	1
Rhbdf2	No	Y	No	Yes(CuffDiff & DESeq2)	1
Unc13d	No	Y	No	No	0
Arhgap23	No	Y	No	Yes(CuffDiff & DESeq2)	0
Ap1s1	No	Y	No	No	0
Tmem18	No	Y	No	No	0
Runx3	No	Y	No	No	0
Exosc9	No	Y	No	Yes(DESeq2)	0
Lrsam1	No	Y	No	No	2
Fam109a	No	Y	No	Yes(DESeq2)	0
Dtnbp1	No	Y	No	No	0
Tada1	No	Y	No	No	2
1500002O20Rik	No	Y	No	No	2
Slc29a3	No	Y	No	Yes(DESeq2)	1
Dhx35	No	Y	No	Yes(DESeq2)	1
Plbd2	No	Y	No	No	0
Zbtb34	No	Y	No	No	1
Pofut2	No	Y	No	No	0
Fam86	No	Y	No	No	1
Med24	No	Y	No	No	0
Rxrb	No	Y	No	No	0
Scfd2	No	Y	No	Yes(DESeq2)	0
Spryd3	No	Y	No	No	0
Stx8	No	Y	No	No	1
1110059E24Rik	No	Y	No	No	0
Cep152	No	Y	No	No	0
4933431E20Rik	No	Y	No	Yes(CuffDiff)	0
Cd37	No	Y	No	No	1
Mcts1	No	Y	No	No	2
Kremen1	No	Y	No	Yes(DESeq2)	1
Pja1	No	Y	No	No	1
Pomp	No	Y	No	Yes(DESeq2)	2
Fbxl18	No	Y	No	No	0
Lphn1	No	Y	No	No	0

Fam55d	No	Y	No	No	1
Sptlc1	No	Y	No	No	1
Satb1	No	Y	No	Yes(CuffDiff)	1
Srebf1	No	Y	No	Yes(CuffDiff & DESeq2)	0
Rgs3	No	Y	No	No	0
Wbp2	No	Y	No	No	0
Flt1	No	Y	No	No	2
Pdzd2	No	Y	No	No	1
Pla2r1	No	Y	No	No	0
Psmd3	No	Y	No	Yes(CuffDiff & DESeq2)	2
Hn1l	No	Y	No	No	1
Syf2	No	Y	No	No	0
Fbrsl1	No	Y	No	No	1
Idh2	No	Y	No	No	1
Zbtb43	No	Y	No	Yes(DESeq2)	1
Bcl9l	No	Y	No	Yes(DESeq2)	0
Zfp710	No	Y	No	No	3
Fam129b	No	Y	No	Yes(DESeq2)	2
Itgb6	No	Y	No	No	2
Cep164	No	Y	No	No	0
Trps1	No	Y	No	No	0
Anxa8	No	Y	No	No	0
Psmd7	No	Y	No	Yes(DESeq2)	0
Aldh1l1	No	Y	No	No	0
Scml4	No	Y	No	No	0
Mapkap1	No	Y	No	Yes(DESeq2)	0
Poldip3	No	Y	No	No	1
Osbpl3	No	Y	No	No	2
Ophn1	No	Y	No	No	0
Bcr	No	Y	No	No	1
Slc2a1	No	Y	No	No	0
Fbn1	No	Y	No	No	0
Tsc1	No	Y	No	Yes(DESeq2)	1
Akt1	No	Y	No	Yes(DESeq2)	0
Tbc1d5	No	Y	No	No	0

Tsc22d2	No	Y	No	No	1
Mapk8ip3	No	Y	No	No	1
Capn5	No	Y	No	No	3
Rcor3	No	Y	No	Yes(DESeq2)	0
Fech	No	Y	No	No	1
Ano6	No	Y	No	No	1
Rgnef	No	Y	No	No	1
Rp2h	No	Y	No	No	1
Nars	No	Y	No	Yes(DESeq2)	1
Ppp1r9b	No	Y	No	Yes(DESeq2)	0
Tshz1	No	Y	No	Yes(CuffDiff & DESeq2)	1
4933407C03Rik	No	Y	No	No	1
Nptn	No	Y	No	No	2
Lamc1	No	Y	No	Yes(DESeq2)	1
Rnu12	No	Y	No	No	1
Cic	No	Y	No	No	0
Gcn1l1	No	Y	No	No	2
Col6a2	No	Y	No	No	1
Wbp7	No	Y	No	No	1
Pcnt	No	Y	No	No	0
Ahctf1	No	Y	No	No	2
Lcor	No	Y	No	No	2
Wac	No	Y	No	No	0
Med13l	No	Y	No	No	2
Mfsd4	No	Y	No	No	0
Foxn3	No	Y	No	No	2
Tsc22d1	No	Y	No	No	1
Anapc1	No	Y	No	No	1
Pbx1	No	Y	No	No	1
Yipf6	No	Y	No	No	0
Txnrd1	No	Y	No	Yes(DESeq2)	1
Elf3	No	Y	No	No	1
Cdc42bpa	No	Y	No	Yes(CuffDiff & DESeq2)	2
Tnrc18	No	Y	No	No	0
Lass4	No	Y	No	Yes(CuffDiff)	0

Ttc14	No	Y	No	No	0
Fndc3a	No	Y	No	Yes(DESeq2)	1
Rplp0	No	Y	No	No	2
Mll2	No	Y	No	No	0
Pebp4	No	N	No	No	1
Fgf16	No	N	No	No	0
Mir139	No	N	No	No	1
Il25	No	N	No	No	1
Tmem8c	No	N	No	No	0
Actrt2	No	N	No	No	1
Mir719	No	N	No	No	0
Gk2	No	N	No	No	0
Mir346	No	N	No	No	0
Mir760	No	N	No	No	1
Samd7	No	N	No	No	2
Adm2	No	N	No	No	0
Fgf8	No	N	No	No	1
Sox3	No	N	No	No	0
Mir383	No	N	No	No	0
2200002J24Rik	No	N	No	No	0
Olfm4	No	N	No	Yes(DESeq2)	1
Gm11202	No	N	No	No	0
Cyp2c29	No	N	No	No	0
Fbxo16	No	N	No	No	0
Cyp2c66	No	N	No	No	0
Fam178b	No	N	No	No	0
C130026L21Rik	No	N	No	No	1
Cacng1	No	N	No	No	2
Fam163b	No	N	No	No	2
Ifna9	No	N	No	No	0
Wbscr28	No	N	No	No	0
Cmtm5	No	N	No	No	1
4930571K23Rik	No	N	No	No	1
Lhx4	No	N	No	No	2
Tekt4	No	N	No	No	1

1700073E17Rik	No	N	No	No	1
LOC16697	No	N	No	No	0
Lrriq4	No	N	No	No	2
Nkx6-2	No	N	No	No	0
Gucy2e	No	N	No	No	2
Prrxl1	No	N	No	No	1
Vgf	No	N	No	No	0
Spaca3	No	N	No	No	0
Mir691	No	N	No	No	0
Gm6484	No	N	No	No	0
B3gat1	No	N	No	No	0
Tmem28	No	N	No	No	1
D030018L15Rik	No	N	No	No	1
Tnfsf4	No	N	No	No	1
Gas2l2	No	N	No	No	0
Nmur2	No	N	No	No	0
A530053G22Rik	No	N	No	No	1
Vmn2r98	No	N	No	No	1
Sds	No	N	No	No	0
Alppl2	No	N	No	No	0
Egr4	No	N	No	No	0
1700096J18Rik	No	N	No	No	0
Oas1e	No	N	No	No	0
Sgcz	No	N	No	No	0
Vmn1r27	No	N	No	No	3
1700026L06Rik	No	N	No	No	1
Slc24a4	No	N	No	No	1
Cyp3a11	No	N	No	No	0
Cdh23	No	N	No	No	0
Kcne1	No	N	No	No	2
Antxrl	No	N	No	No	1
